# Cost-Effectiveness of “Golden Mustard” for Treating Vitamin A Deficiency in India

**DOI:** 10.1371/journal.pone.0012046

**Published:** 2010-08-10

**Authors:** Jeffrey Chow, Eili Y. Klein, Ramanan Laxminarayan

**Affiliations:** 1 School of Forestry and Environmental Studies, Yale University, New Haven, Connecticut, United States of America; 2 Department of Ecology and Evolutionary Biology, Princeton University, Princeton, New Jersey, United States of America; 3 Center for Disease Dynamics, Economics, and Policy, Washington, D. C., United States of America; 4 Princeton Environmental Institute, Princeton University, Princeton, New Jersey, United States of America; Canadian Agency for Drugs and Technologies in Health, Canada

## Abstract

**Background:**

Vitamin A deficiency (VAD) is an important nutritional problem in India, resulting in an increased risk of severe morbidity and mortality. Periodic, high-dose vitamin A supplementation is the WHO-recommended method to prevent VAD, since a single dose can compensate for reduced dietary intake or increased need over a period of several months. However, in India only 34 percent of targeted children currently receive the two doses per year, and new strategies are urgently needed.

**Methodology:**

Recent advancements in biotechnology permit alternative strategies for increasing the vitamin A content of common foods. Mustard (*Brassica juncea*), which is consumed widely in the form of oil by VAD populations, can be genetically modified to express high levels of beta-carotene, a precursor to vitamin A. Using estimates for consumption, we compare predicted costs and benefits of genetically modified (GM) fortification of mustard seed with high-dose vitamin A supplementation and industrial fortification of mustard oil during processing to alleviate VAD by calculating the avertable health burden in terms of disability-adjusted life years (DALY).

**Principal Findings:**

We found that all three interventions potentially avert significant numbers of DALYs and deaths. Expanding vitamin A supplementation to all areas was the least costly intervention, at $23–$50 per DALY averted and $1,000–$6,100 per death averted, though cost-effectiveness varied with prevailing health subcenter coverage. GM fortification could avert 5 million–6 million more DALYs and 8,000–46,000 more deaths, mainly because it would benefit the entire population and not just children. However, the costs associated with GM fortification were nearly five times those of supplementation. Industrial fortification was dominated by both GM fortification and supplementation. The cost-effectiveness ratio of each intervention decreased with the prevalence of VAD and was sensitive to the efficacy rate of averted mortality.

**Conclusions:**

Although supplementation is the least costly intervention, our findings also indicate that GM fortification could reduce the VAD disease burden to a substantially greater degree because of its wider reach. Given the difficulties in expanding supplementation to areas without health subcenters, GM fortification of mustard seed is an attractive alternative, and further exploration of this technology is warranted.

## Introduction

Vitamin A deficiency (VAD) is an important nutritional problem in large parts of the developing world, affecting as many as 130 million children [1]. VAD results in an increased risk of severe morbidity and mortality due to anemia and depressed resistance to infectious disease and is responsible for more than a million child deaths annually. The most apparent result of VAD is clinical eye symptoms due to xerophthalmia (a destructive dryness of the conjunctival epithelium, manifested in early stages by deposits in the conjunctiva known as Bitot's spots), making it the leading cause of preventable childhood blindness in developing countries [1]. VAD also affects many women in poor countries, specifically causing night blindness, anemia, and increased maternal morbidity and mortality during pregnancy and lactation.

**Figure 1:**
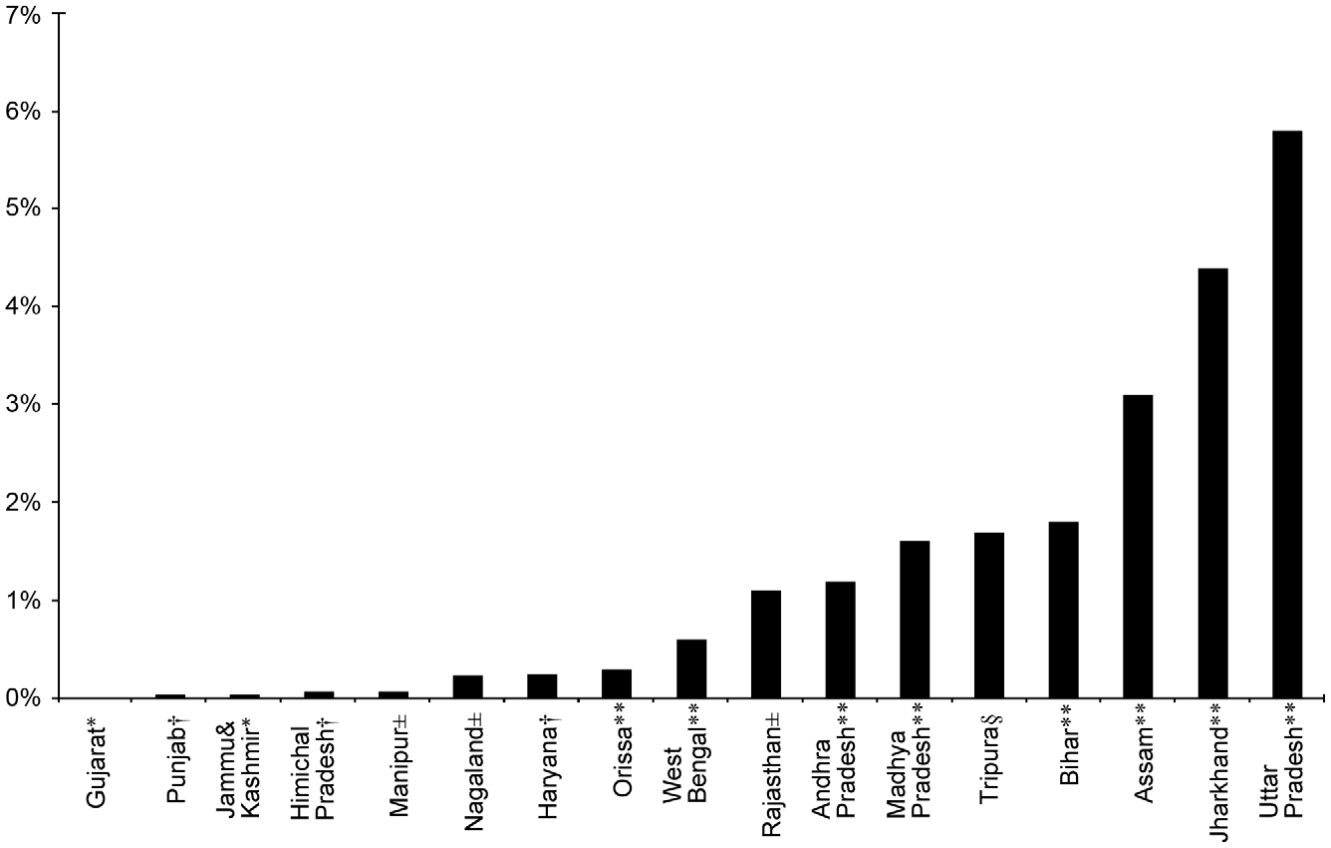
Prevalence of Bitot's spots among children in 17 Indian states. Sources: * [42]; ** [5]; ± [59]; † [60]; § [61].

Though the incidence of clinical VAD in India has declined significantly over the past few decades, the country has the greatest number (more than 35 million) and the greatest percentage of VAD children in the world [1], and VAD persists as a public health problem, especially in rural areas [2], [3]. The overall prevalence of xerophthalmia among children is 1.7 percent [4], and approximately 0.8 percent of all children suffer from Bitot's spots [5], but state and local rates can vary significantly (Figure 1). Subclinical VAD is even more prevalent, with recent estimates (31–57 percent of children under six) placing India among the highest in the world [1], [4]. VAD also afflicts more than 12 percent of all mothers nationwide [6], and 19 states have night blindness rates for pregnant women exceeding 5 percent [7].

VAD occurs primarily in the lower socioeconomic strata of poor countries, among people with limited food choices and diets predominated by less nutritious staple foods [8]. Strategies for addressing VAD have traditionally fallen into three main categories: supplementation, food fortification, and diet and behavior modification. Recent advancements in biotechnology have created an additional avenue for addressing VAD: biofortification—using genetic recombinant technology to develop genetically modified (GM) fortified products with enhanced expression of provitamin A carotenoids, such as vitamin A–enhanced rice, or “golden rice” [9]. It has been suggested that introducing golden rice in India could decrease VAD prevalence by as much as 59 percent [10], but people in some regions in India with a high prevalence of VAD traditionally do not consume significant amounts of rice. Moreover, the unconventional color of golden rice may discourage its consumption, and poor people may not consume enough fat to facilitate biological absorption of the carotenoids [11]–[13].

However, rice is not the only crop that has been modified to express high levels of beta-carotene; mustard (*Brassica juncea*) can be modified to express significantly higher levels of beta-carotene, as much as 600 µg/g [14], [15]. In addition, beta-carotene levels are conserved when the mustard seeds are pressed into oil, which is widely consumed in many parts of India with a high prevalence of VAD, even by lower socioeconomic groups (see Supplementary Notes S1 and Supplementary Figures S1 and S2 for more information on mustard oil consumption in India). Only a few drops of the transgenic oil would satisfy the recommended daily allowance for vitamin A intake.

Periodic, high-dose vitamin A supplementation is the most widely used public health approach to preventing deficiency [16]. A single dose can provide a reserve of vitamin A to compensate for reduced dietary intake or increased need over a period of several months. However, in India the percentage of targeted children currently receiving two doses per year, distributed by the Reproductive and Child Health program, is reported to be approximately 30 percent [6], [17], and new strategies are urgently needed. We examined the costs and health benefits of GM fortification of mustard oil in India. Although several recent studies have examined the possible effect of different genetically modified foods [10], [13], [18], [19], none have compared the costs and outcomes of different approaches. Thus, in our cost-benefit analysis we include three population-based strategies to improve the vitamin A status of at-risk groups: high-dose vitamin A supplementation for preschoolers (ages 1 to 4); industrial fortification of mustard oil during processing; and GM fortification of mustard oil.

Our analysis focused on states in India that consume mustard oil in significant quantities and that have been shown to have a high prevalence of VAD. These include states where mustard is grown and consumed (Uttar Pradesh, Bihar, Assam, West Bengal, Madhya Pradesh, Rajasthan, Jharkhand, Orissa, Haryana, Gujarat, Punjab, Jammu and Kashmir, and Himachal Pradesh) and states where mustard is not grown but consumed (Manipur, Nagaland, and Tripura). We also emphasized those states where Bitot's spots prevalence rates indicate that VAD is a public health problem: Uttar Pradesh, Bihar, Assam, West Bengal, Madhya Pradesh, Rajasthan, Jharkhand, and Tripura. These analyses focused on the rural areas of these states, where the vast majority of the population lives. Information on prevalence rates for urban areas is not as comprehensively available as those for rural areas, where surveyors such as the National Nutrition Monitoring Bureau (NNMB) focus their efforts. Hence, we included a subset of states with urban prevalence estimates (Uttar Pradesh, Bihar, West Bengal, Madhya Pradesh, Jharkhand, Jammu and Kashmir) as well as the urban populations in two well-studied metropolitan areas, Chandigarh and Delhi, for urban-specific analyses. The implementation of health initiatives in India is typically the responsibility of individual states, and it would make little sense for states with low prevalence of VAD or negligible consumption of mustard oil to participate in vitamin A GM fortification efforts.

## Methods

We examined alternative population-based strategies to alleviate VAD by calculating the avertable health burden in terms of disability-adjusted life years (DALY) as well as avertable deaths and by comparing incremental cost-effectiveness ratios (ICER) in terms of unit cost per both DALY and death averted. DALYs are a measure of healthy life years—the sum of the present value of years of future lifetime lost through premature mortality plus the present value of years of future lifetime, adjusted for the average severity of the mental or physical disability caused by a disease or injury [20]. The DALY metric allows comparisons of alternative health strategies using a single index that combines information about mortality and morbidity and has been widely used in similar analyses [10], [13], [18], [19].

We also calculated the effect of a specific intervention as the number of DALYs averted, defined as
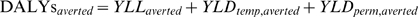
(1)where Y*LL_averted_* is the number of discounted life-years averted, and Y*LD*_temp,averted_ and Y*LD*_perm,averted_ are years of life with temporary and permanent disability averted, respectively, due to morbidity. To account for varying disease levels among the different target groups affected by the three strategies, Y*LL*_averted_, Y*LD*_temp,averted_, and Y*LD*_perm,averted_ are defined as follows:
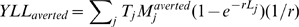
(2)

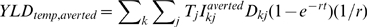
(3)

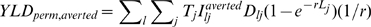
(4)where *T_j_* is the total number of people in the target group *j*, 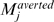 is the reduction in the mortality rate of the target group due to the intervention and *L_j_* is the average remaining life expectancy. 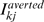 and 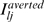 are reductions in the morbidity rates of temporary and permanent sequelae *k* and *l*, respectively. *D_kj_* and *D_lj_* are the corresponding disability weights. The parameter *t* is the duration of the intervention, and *r* is the discount rate of future life years, which is the standard 3 percent used in DALY calculations as recommended by the World Bank [21].

We considered the effect of an intervention over a 20-year time frame. We assumed that the reduction of temporary sequelae, such as Bitot's spots and night blindness, is impermanent, lasting only as long as the duration of the intervention. For example, an individual receiving vitamin A supplementation up to an age of 4 years could still develop Bitot's spots at age 5, at which point the intervention would have ceased for that individual. However, permanent sequelae, such as blindness, are averted for the lifetime of the treated individual, since the intervention allows an individual to pass through the most high-risk period for the onset of blindness unscathed. Second, we assumed that individuals whose lives have been saved by the intervention go on to live for their remaining life expectancy. Finally, we assumed that an intervention lasting less than two years for a cohort of preschool children would reduce neither the mortality rate nor permanent sequelae among that cohort, since a single dose compensates for reduced intake for only several months. Thus, we conservatively assume that premature cessation of supplementation would put a child at full risk of VAD morbidity and mortality.

We evaluated the cost-effectiveness of a particular intervention relative to other possible interventions according to the ratio of its total cost to its total effectiveness over the entire 20-year time frame, assuming that the program is already running at full capacity. Costs were discounted at a 3 percent annual rate. Effectiveness is in terms of averted deaths or averted DALYs. Because we lacked baseline data by state on current supplementation coverage, and anecdotal accounts suggest it is lower than reported [22], we assumed that VAD data were collected in the absence of an intervention. Hence, our baseline assumes that little or no intervention currently exists, but we account for possible underlying supplementation coverage by applying a range of effectiveness rates. All ICERs express the incremental impact of each non-dominated intervention. A larger cost-effectiveness ratio indicates a higher cost per unit of health gained. Prior to the calculation of ICERs, we excluded interventions that are strictly dominated—that is, interventions that are both less effective and more costly than at least one other strategy. In addition to calculating the number of DALYs averted for each intervention, we also calculated ICERs for deaths averted. Lastly, we attributed a monetary value to a DALY to calculate internal rates of return (IRR). The monetary value chosen was annual per capita income for the year 2003–2004 [23]. As with similar analyses [13], the IRR estimates generated should be interpreted carefully. The goal of this approach was to estimate a useful metric for economic analysis rather than to predict rates of return on investment. These metrics offer an index, scaled to the average local living standard, by which the comparative value of an intervention can be judged.

Finally, GM-fortified mustard is still in the research and development stage. Hence, in this *ex ante* framework, parameters such as program costs and the reductions in mortality and morbidity rates are not yet known and must be estimated based on assumptions about health services, agriculture, and food production and delivery systems in India. All assumptions are based on the best biomedical research available, drawn from published studies as well as interviews with experts in VAD and mustard production and consumption in India.

### Data

Our analysis used state-specific data where possible to estimate state-specific mustard oil consumption among young children and women of reproductive age, as well as rates of effectiveness in reducing morbidity and mortality associated with vitamin A deficiency. We calculated all results in 2005 U.S. dollars, using the exchange rate of Rs. 40 = US$1.

### Strategies

We analyzed three interventions: (1) high-dose vitamin A supplementation; (2) industrial fortification of mustard oil; and (3) GM fortification of mustard oil with genetically modified mustard seed. Averted DALYs were calculated based on (1) avoided disability due to clinically apparent VAD morbidity and (2) avoided mortality due to subclinical and clinical VAD. As in other analyses [13], averted morbidity includes cases of Bitot's spots and night blindness during the intervention, as well as lifetime disability due to blindness. Bitot's spots and night blindness are treated as temporary diseases, the durations of which are taken at one year to avoid inflation of DALYs. For blindness, we conservatively assumed that the prevalence of corneal xerophthalmia cases is one-tenth the prevalence of Bitot's spots [24]–[29], and without treatment, one-half of all corneal cases lead to blindness [16]. Averted morbidity DALYs were calculated as the sum of disability due to Bitot's spots, night blindness, and blindness multiplied by their respective treatment effectiveness rates. Because data on prevalence rates are scant, other sequelae of xerophthalmia due to VAD, such as corneal xerosis and keratomalacia, are not considered. Consequently, estimates for averted cotemporaneous disability are conservative.

#### High-dose vitamin A supplementation

We considered a massive dose supplementation program providing semiannual doses of 200,000IU to preschoolers, who are more responsive to high-potency vitamin A supplementation and more susceptible to corneal xerophthalmia [16]. It has been shown that vitamin A supplementation does not benefit early infant survival [30]–[33]. The current vitamin A supplementation program in India concentrates on children 9 to 36 months [34].

Because we lacked baseline data on the effectiveness of vitamin A supplementation to reduce morbidity specific to our study areas, we used a range of values reported in the literature (Table 1). Costs were calculated by multiplying the number of preschool children by per capita costs (Table 2). The extent of mortality reduction due to vitamin A supplementation is somewhat contentious, ranging from estimates as high as 23 percent [35], [36] to insignificant [24], [37], [38]. A recent study from northern India found reductions in mortality of about 4 percent [39]. Thus, we assumed a range of 4 to 23 percent. Avoided mortality was converted to DALYs by multiplying the number of deaths averted by life expectancy. The National Prophylaxis Programme for Prevention of Blindness due to Vitamin A Deficiency in India is implemented through the existing Primary Health Care infrastructure [34]. Village-level health subcenters, each serving a population of 5,000 (3,000 in tribal, hilly, or inaccessible areas), conduct vitamin A supplementation. Drawing from state subcenter data [40], we estimated subcenter coverage by conservatively assuming each subcenter covers 3,000 people and then dividing the number of people covered by the total state population. Because village-level health subcenters serve only a fraction of the population in each state, we included supplementation costs for all areas currently served by health subcenters [40] as well as the additional costs of supplementing children in areas currently lacking subcenter coverage, which would require additional resources for delivery [22]. The supplementation coverage achievable was adjusted downward according to an estimated rate of health worker absenteeism [41]. Given our assumptions regarding coverage and absenteeism, we found that state coverage ranged from 20 percent in Uttar Pradesh to 61 percent in Himachal Pradesh, with an average of 25 percent coverage across our entire study area. However, our estimates did not consider the costs for expansion, program infrastructure, or other fixed costs, and we assumed that the effectiveness rates were uncorrelated with subcenter coverage.

**Table 1:** Effectiveness of interventions in reducing VAD burden.

Intervention	Percentage reduction
Bitot's spots (children)	26–75
Night blindness (children)	46–100
Blindness (children)	43–75
Mortality (children)	4–23

**Table 2:** Cost of each intervention.

Intervention	Cost
**Supplementation**	
Shipping, storage, delivery, and wastage for 2 doses of vitamin A per year	US$0.07 (Rs. 2.58) per child per year [62]
Administrative and regulatory costs, training, promotional and educational materials, and program monitoring and evaluation	US$ 0.08 (3.33Rs) per child per year [63]
Total (areas w/subcenters)	US$0.15 (Rs. 5.91) per child per year
Total (areas w/o subcenters)	US$0.65 (Rs. 26) per child per year [22]
**Fortification**	
Cost to fortify 1 kg of oil	US$0.001 (Rs. 0.045) to US$0.005 (Rs. 0.22) [63], [64]
Administrative and regulatory costs, training, promotional and educational materials, and program monitoring and evaluation (per kg of oil)	US$0.002 (Rs. 0.062) [63]
Bottling costs (per l of oil)	US$0.09 (Rs. 3.75) [65], [66]
**GM fortification**	
Administrative and regulatory costs, training, promotional and educational materials, and program monitoring and evaluation (per kg of oil)	US$0.002 (Rs. 0.062) [63]
Bottling costs (per l of oil)	US$0.09 (Rs. 3.75) [65], [66]
One-time regulatory approval cost	US$5.6 million (22.7 crore Rs.) [66]

#### Industrial fortification of mustard oil with vitamin A

Avertable morbidity and mortality were considered not just among preschool children but also among new and expectant mothers because broad-based fortification of a commonly consumed food item, such as mustard oil, would increase the VA intake of the entire population. Population estimates for new and expectant mothers for a given year are based on age-specific fertility rates and female population numbers from each state.

We calculated morbidity and mortality efficacy based on current vitamin A consumption, improved vitamin A (IVA) intake with fortified mustard oil, and recommended daily allowances (RDA), following methods used by Zimmermann and Qaim [13]. RDA values for preschool children and for new and expectant mothers were estimated to be 400µg and 775µg, respectively [3]. IVA is calculated by adding current vitamin A consumption [3], [42] to current mustard oil consumption times a retinol concentration of 18µg/g and a VA retention rate. Retention rates of VA supplement during storage exceed 92 percent after six months [43]. VA supplement has a retention rate of 78–90 percent after brief, low-heat frying, which is how approximately 90 percent of oil consumed is used [43]. Because the supply chain from producer to retailer is fairly efficient, and because rural households typically use mustard oil soon after purchase, we chose a conservative retention rate of 80 percent. Mustard oil consumption among preschoolers is estimated based on average consumption data (kg/month/person/household) [44], state-specific household age structure, and age-specific consumption rates [44], [45] and conservatively scaled down based on income and age because the poorest segments of a population tend to consume less mustard oil per person than average [44] and children consume less than adults.

DALYs from averted morbidity and mortality were calculated under the assumption that fortified mustard is available in the same proportion for all consumers, and that for new and expectant mothers the intervention prevents death among a different set of women each year. The costs included the cost for the fortification process itself as well as other costs to prevent vitamin A degradation and ensure product quality, such as bottling and quality control (Table 2). We assumed that all mustard oil consumed is required to be fortified, and we explicitly accounted for administrative and regulatory costs, training, promotional and educational materials, and program monitoring and evaluation. Because vitamin A fortificant degrades readily when exposed to light, the costs of bottling in opaque containers was also considered.

#### Biofortification with genetically modified mustard

Since GM fortification and industrial fortification are similar delivery strategies for vitamin A, the cost-effectiveness analyses of the two interventions were alike except for parameter adjustments regarding costs and vitamin A content (Table 2). Based on field trials, in which pressed oil from genetically modified mustard seed could contain as much as 600µg per gram of oil, we conservatively assumed that GM-fortified mustard oil has a beta-carotene content of 185µg/g [46], and that only 71 percent of the beta-carotene is retained after cooking [47]. The variety of mustard being developed for expression of beta-carotene accounts for 70–80 percent of the mustard grown in India. Hence, we assume that only 75 percent of the mustard seed that is pressed into oil is GM fortified. Based on these assumptions, we calculated an effective concentration of 49.3µg of vitamin A per g of oil at the consumption level.

Costs related to the distribution, sale, and regulation of GM-fortified oil were assumed to be the same as for industrially fortified oil, since both would require bottling in opaque containers and presumably require similar regulatory, monitoring, and enforcement frameworks. However, there are no costs related to adding artificial fortificant, since beta-carotene would occur naturally in GM-fortified mustard seeds and the cold-pressed oil produced from it. Instead, we incorporated a one-time fixed cost of US$5.6 million (Rs. 22.7 crore) to account for the costs of licensing on the assumption that the R&D has already been completed and the regulatory mechanisms for licensing such products are already in place. We apportioned this amount among the states considered in this analysis according to their total rural population size and applied the same rate to urban populations. We estimated that these fixed costs would amount to approximately US$0.01 (Rs. 0.40) per person.

To prevent the escape of engineered genetic material into natural ecosystems, the growing of genetically modified mustard on a commercial scale could require bioconfinement practices by farmers, as well as additional regulatory and monitoring activities [48]. However, cost estimates for regulation of transgenic crops in India do not yet exist because the policies themselves are still being formulated, and there is a great deal of regulatory ambiguity regarding transgenic crops. The most comparable example is *Bt* cotton, which in 2002 became the first transgenic crop approved for cultivation in India [49]. Farmers and others involved in cotton production do not yet incur additional costs for producin*g Bt* cotton other than a premium for the purchase of transgenic seeds. Since GM-fortified mustard is not expected to be sold at an additional premium and because the regulatory framework is still unclear, we ignored any additional costs of growing GM-fortified versus regular mustard.

### Sensitivity analysis

Because of the uncertainty surrounding our assumed parameters, we used a Monte Carlo sampling method to evaluate the robustness of our results [50]. We found that our assumed mortality range had a significant effect on the results. To more accurately depict the range over which our results varied, we adjusted the analysis to examine an optimistic scenario and a conservative scenario. In the optimistic scenario we assumed a mortality reduction rate of 23 percent, and in the conservative scenario we assumed a mortality reduction rate of 4 percent. All other parameters were varied over their full range to produce confidence intervals around our different scenarios (see the Supplementary Notes S2 for detailed information on sensitivity analysis and Table S1 for parameter ranges).

## Results

Our analysis estimated the effectiveness in reducing morbidity and mortality associated with vitamin A deficiency through three methods: supplementation for 1- to 4-year-olds, industrial fortification of mustard oil, and GM fortification of mustard seed. Each scenario was evaluated using a conservative efficacy rate (4 percent) and an optimistic efficacy rate (23 percent) of averted mortality, and results were obtained for the number of DALYs and deaths averted over a 20-year time frame. The costs of each intervention are presented in Table 3, and the incremental cost-effectiveness of each non-dominated intervention in $/DALY and $/Death Averted are presented in Tables 4 and 5, respectively. The ICER of moving from one intervention to a more expensive alternative is the difference in cost divided by the difference in DALYs or deaths averted associated with each intervention. We also calculated an internal rate of return for each intervention in Table 6, though these are not incremental.

**Table 3:** Cost of intervention implementation.

Intervention	Cost US$ (millions)
Supplementation	637 (607–667)
*areas w/subcenters*	36 (35–38)
*areas w/o subcenters*	600 (570–630)
Fortification	3,177 (3,099–3,255)
GM fortification	3,103 (3,028–3,179)

Note: parentheses denote 95 percent confidence intervals.

**Table 4:** DALYs averted for each intervention over 20-year time horizon.

Intervention	DALYs averted (millions)		Incremental cost-effectiveness ($/DALY)	
	*Low efficacy*	*High efficacy*	*Low efficacy*	*High efficacy*
Supplementation	12.7 (12.4–13.1)	27.6 (27.3–28.0)	50 (46–54)	23 (22–24)
Fortification	10.8 (10.5–11.1)	18.6 (18.2–19.0)	Dominated	Dominated
GM fortification	18.1 (17.8–18.5)	33.7 (33.2–34.1)	450 (438–461)	403 (389–418)

Note: Parentheses denote 95 percent confidence intervals. Each intervention is compared with immediately less effective and non-dominated alternative intervention.

**Table 5:** Deaths averted for each intervention over 20-year time horizon.

Intervention	Deaths averted (thousands)		Incremental cost-effectiveness ($/death averted)	
	*Low efficacy*	*High efficacy*	*Low efficacy*	*High efficacy*
Supplementation	105 (102–109)	608 (604–611)	6,100 (5,600–6,600)	1,000 (1,000–1,100)
Fortification	86 (84–88)	356 (349–362)	Dominated	Dominated
GM fortification	113 (110–116)	654 (647–660)	303,300 (310,000–297,100)	53,000 (48,700–57,900)

Note: Parentheses denote 95 percent confidence intervals. Each intervention is compared with immediately less effective and non-dominated alternative intervention.

**Table 6:** Internal rates of return for each intervention.

Intervention	Internal Rate of Return	
	*Low efficacy*	*High efficacy*
Supplementation	68 (65–70)	104 (102–107)
*areas w/subcenters*	142 (139–145)	195 (192–198)
*areas w/o subcenters*	59 (56–61)	93 (90–95)
Fortification	6 (5–7)	22 (21–22)
GM fortification	22 (21–23)	43 (42–44)

Note: Parentheses denote 95 percent confidence intervals. Internal rates of return are not incremental.

As shown in Table 4, we estimate that over a 20-year period, GM fortification could avert the most DALYs (18 million–34 million) and deaths (113,000–654,000), but at approximately 5 times the cost of supplementation, which could avert 12 million–28 million DALYs and 105,000–608,000 deaths. The incremental cost of supplementation, evaluated at $23 (95 percent CI: 22–24) to $50 (95 percent CI: 46–54) per DALY averted, is far less than the incremental cost of GM fortification, $403 (95 percent CI: 389–418) to $450 (95 percent CI: 438–461) per DALY averted. A similar relationship occurs when considering only deaths averted (Table 5). However, these results ignore any fixed costs of expanding supplementation services to populations without subcenters. Moreover, supplementation targets only young children and does not alleviate the suffering of pregnant mothers. Industrial fortification averts fewer DALYs and deaths at a greater cost than both GM fortification and supplementation and thus is dominated by both of them. Industrial fortification is strictly dominated by GM fortification because it is assumed to have a lower vitamin A content than GM-fortified oil.

The cost-effectiveness of supplementation varies depending on health subcenter coverage. In areas where health subcenters exist, the cost per DALY averted can be as low as $5 to $11 (or $200 to $1,300 per death averted), whereas additional delivery costs necessary in areas without subcenters result in an incremental cost-effectiveness ratio of $42 to $90 per DALY averted (or $1,900 to $11,100 per death averted). Of the DALYs avertable via supplementation, 9.5 million (95 percent CI: 9.2–9.8) to 20.6 million (95 percent CI: 20.3–20.8) occur in areas lacking subcenter coverage, versus 3.2 million (95 percent CI: 3.1–3.3) to 7.1 million (95 percent CI: 7.0–7.2) in areas with existing subcenters. Similarly, the number of avertable deaths is greater, 78,000 (95 percent CI: 75,000–81,000) to 450,000 (95 percent CI: 447,000–453,000), in areas without subcenters than in areas with subcenter coverage, 27,000 (95 percent CI: 26,000–28,000) to 158,000 (95 percent CI: 157,000–158,000).

The cost-effectiveness ratio for expanding supplementation to all areas translates to an internal rate of return of 68–104 percent, which far exceeds the internal rate of return of industrial fortification (6–22 percent) and GM fortification (21–42 percent) (Table 6). The internal rate of return for supplementation in areas where health subcenters are located (142–195 percent) is greater than the IRR in areas without subcenters (59–93 percent), again because of greater delivery costs in those areas.

Estimates for DALYs and deaths averted for each intervention exhibited significant variation among states and between rural and urban areas (see Supplementary Table S2), but overall patterns remained the same as those described above. VAD interventions had higher cost-effectiveness ratios across all interventions in states that had low underlying mortality rates, such as West Bengal and Himachal Pradesh. Interventions also had higher cost-effectiveness ratios in urban areas because of their lower baseline mortality rates.

## Discussion

Vitamin A supplementation, at US$23–$50 per DALY averted, is much less costly than either fortification intervention. Because of the substantially greater cost for GM fortification, the incremental cost-effectiveness of this intervention is US$405–$450 per DALY averted, though it would reach a wider swath of the population, particularly pregnant mothers. Industrial fortification of mustard oil, which averts fewer DALYs and costs more than either supplementation or GM fortification, is not an attractive option for policymakers under the assumptions we have made about costs and benefits.

Our estimates for the cost-effectiveness of a vitamin A supplementation program targeting preschool children are in line with estimates previously reported in the literature, at least for areas with subcenter coverage [51], [52]. However, our cost-effectiveness estimates for people without access to functioning health subcenters, a situation that applies to some 70 percent of the population, are higher than these previous estimates. The difference may be due to the numerous assumptions in our analysis intended to err on the conservative side of avertable disease burden, as well as uncertainties about the cost of supplementation. In particular, despite recent evidence that VAD supplementation is effective in significantly reducing morbidity [36], our lack of data on existing levels of supplementation required us to apply conservative effectiveness rates, since the incremental effectiveness of any intervention would be expected to diminish with any underlying level of supplementation greater than zero.

Based on the few formal cost-effectiveness studies of vitamin A fortification comparable to ours, fortifying oil in India may be more costly than interventions in other countries that use different vitamin A delivery vectors. For example, in Central America the annual cost of sugar fortification has been estimated at around US$1 per high-risk person reached (i.e., young children and women of childbearing age) [53], [54], and a study of wheat fortification in the Philippines indicated an annual cost of $1.3 to $2.3 per child with inadequate vitamin A intake [55]. These reported costs are less than our estimate for fortification of approximately $4.50 per year per person at risk (i.e., pregnant women and children). Such differences may be at least partly related to India's large number of small, disaggregated mustard oil producers—in contrast to the concentrated manufacture of fortification vehicles evaluated in other studies—and the consequent additional difficulty and cost of ensuring that the oil is fortified.

Other analyses of GM fortification have tended to find that introducing biofortified products may be less costly than our estimates for GM mustard. In an *ex ante* analysis of golden rice in the Philippines, Zimmerman and Qaim [13] estimated that introducing GM-fortified rice would result in a gain in DALYs of 15,000–85,000 per annum, which is significantly less than the effectiveness of GM mustard, even when accounting for the difference in population size. The expected cost of GM rice, including R&D costs, was estimated to be $10.7 million, with a continuing cost of $0.5 million per annum. Over a 20-year time frame with a 3 percent discount rate, this suggests a total cost of approximately $18.6 million. This cost is significantly less than our estimates for GM mustard, but unlike GM mustard, the estimates for GM rice assumed no significant additional costs associated with distribution. Zimmerman and Qaim estimated an IRR of 66–133 percent, which is greater than our estimates for GM fortification of mustard oil in India. This disparity is partly due to the significantly higher per capita income assumed for the Philippines—$1,030 versus the average $344 assumed in our analysis. Stein and colleagues [10], using new estimates for the vitamin A content of GM rice, estimated that introducing Golden Rice could result in a gain of 204,000–1,382,000 DALYs per annum, which is similar to our calculation for GM mustard. However, they estimated that the total costs for introducing Golden Rice in India, including development, breeding, regulation, dissemination, marketing, and maintenance efforts, would amount to only $16.5 million–$21.5 million over 20 years. Using the same framework, Meenakshi and collegues [18] evaluated the prospective introduction of a number of GM-fortified crops into several countries and also found generally lower costs and less effectiveness, though the range was great.

In contrast to our finding that supplementation was more cost-effective than fortification, other studies comparing alternative vitamin A interventions have found that fortification can be the more attractive option [53], [55], [56]. However, these studies generally acknowledged that supplementation is more appropriate in settings where the fortified food product is not consumed in sufficient amounts. Furthermore, although GM fortification may have a lower cost-effectiveness ratio than supplementation in some cases, it may not deliver adequate amounts of vitamin A and may be insufficient as a stand-alone strategy [11]. Hence, the optimal decision depends on the consumption patterns of the fortified food, and the two strategies can be complementary. For instance, even though our results suggest that supplementation has a substantially lower cost-effectiveness ratio, implementation of a universal supplementation program in India has been problematic. After 30 years, the National Prophylaxis Programme for Prevention of Blindness has achieved coverage of only about 30 percent [6], [17], [22], and controversy over its implementation and effectiveness [57] has further complicated progress.

GM fortification, on the other hand, offers some advantages over supplementation even if the incremental cost-effectiveness ratio is large. Unlike periodic supplementation, GM fortification would provide a continual source of vitamin A and is thus more likely to result in a sustained rise in serum retinol levels [27], [58], providing sustained protection from anemia and infectious disease. Additionally, our analysis shows that GM fortification of mustard oil might avert a substantially greater disease burden than supplementation alone, mostly because it would benefit the entire population, rather than a single targeted age group. Also, seed production and distribution in India are relatively centralized, facilitating implementation and enforcement. However, GM fortification faces some challenges. Although mustard oil is a good vehicle for supplying beta-carotene in states where mustard oil is widely consumed, people in many parts of the country do not consume mustard oil.

Introduction of GM fortified seed may also face acceptance hurdles. Our discussions with farmers suggest that although the introduction of GM-fortified seed would be readily accepted, the color of the resulting oil may face some resistance from consumers, necessitating additional costs for education and awareness campaigns. There also may be regulatory hurdles to widespread use of GM technologies.

In summary, our analysis suggests that vitamin A supplementation is a less costly method of improving the vitamin A status of vulnerable populations in India but would avert fewer DALYs and deaths than fortification via genetically recombinant mustard oil. GM mustard could reduce VAD disease burden by a substantially greater degree because of its potentially wider reach. However, our analysis does not include the fixed costs associated with expanding supplementation in areas currently without subcenter coverage. Our results can help inform the selection of VAD reduction strategies at the state level. They indicate that GM fortification of mustard oil could be used to reach vulnerable populations, especially those that do not readily benefit from supplementation programs, such as mothers and populations without access to Primary Health Care facilities. The optimal approach to addressing VAD in India thus will likely include a mix of strategies, given their relative advantages and disadvantages and the cultural and socioeconomic variations that exist across India. In mustard-consuming states at least, GM fortification of mustard oil has the potential to substantially reduce VAD. States that do not consume mustard oil, however, must continue to rely on supplementation to improve their VA status or develop alternative fortification vehicles.

## Supporting Information

Notes S1 Mustard oil consumption in India.
(0.03 MB DOC)

Notes S2 Sensitivity analysis.
(0.04 MB DOC)

Figure S1 Total domestic consumption of edible oil, 1972–2006, by type. Note: Consumption for 2006 is an estimate. Source: United States Department of Agriculture 2006.
(0.41 MB TIF)

Figure S2 Monthly per capita purchases of mustard oil by household income in rural and urban households of Rajasthan, Uttar Pradesh, Bihar, and Madhya Pradesh, 2005. Source: IMRB International (2006).
(2.84 MB TIF)

Table S1 Parameter values for sensitivity analysis.
(0.11 MB DOC)

Table S2 State-specific cost-effectiveness calculations ($/DALY averted).
(0.05 MB DOC)
