# Supplementary material for: Cost-Effectiveness of “Golden Mustard” for Treating Vitamin A Deficiency in India
Source: PLoS One. 2010 Aug 10;5(8):e12046. doi: 10.1371/journal.pone.0012046 (PMC2919400; doi:10.1371/journal.pone.0012046)
Supplement: Notes S1 — Mustard oil consumption in India. (0.03 MB DOC) [file pone.0012046.s001.doc]

SUPPLEMENTARY NOTES

for

**Cost-Effectiveness of “Golden Mustard” for Treating**

**Vitamin A Deficiency in India**

Jeffrey Chow

Yale University

School of Forestry and Environmental Studies

New Haven, CT, USA

Eili Y. Klein

Princeton University

Department of Ecology and Evolutionary Biology

Princeton, NJ, USA

Ramanan Laxminarayan

Center for Disease Dynamics, Economics, and Policy

Washington, DC, USA

&

Princeton University

Princeton Environmental Institute

Princeton, NJ, USA

# Mustard Oil Consumption in India

Mustard-rapeseed oil has long been one of the most consumed edible oils in India and currently is third, after palm and soybean oil (Figure S1), even though most mustard is consumed only in the north. Consumption of mustard oil has grown about 5 percent per year for the past 40 years and currently stands at approximately 2.3 million metric tons annually. Across many northern states, average consumption exceeds 30 grams per person per month, but this statistic masks both large differences between states and differences within them. Differences in state averages can vary by as much as one-fold in states that are fairly close to each other, and differences within states occur between rural and urban consumers, as well as between different classes (Figure S2). The pattern of consumption across classes suggests that mustard oil is a fairly integral part of many Indian households, regardless of income, though at higher income levels there seems to be a switch to more expensive oils [1].

Although consumption of mustard oil is typically more prevalent in rural areas, total consumption is usually higher in urban areas. In addition, urban households are more likely to use branded oil, and rural households are more likely to consume unbranded (loose) oil. This is largely because branded oils cost around Rs. 60–70 per kg, while loose oil costs from Rs. 48–50 per kg. Despite being called the “poor man’s oil,” mustard oil is roughly double the cost of other oils, such as palm, ricebran, or soybean. This higher price, combined with its pungency, also makes it fairly easy for producers to adulterate it with other, less costly oils. The government has made a push toward more packaged oil since 1998, when loose mustard oil was adulterated with argemone, a poisonous additive, causing a number of illnesses [2].

Most mustard oil (around 90 percent) is used for cooking, and the rest is used for pickling and consumption as raw oil (*sareson-ka-thal*, “oil of mustard”) directly on foods. In rural areas, food is cooked at fairly low heat to maintain the pungency of the oil, while in urban areas, pungency is less desirable and food is cooked at higher temperatures. This is important because studies on red palm oil, soybean oil, and vanaspati indicate that the primary determinants of how much vitamin A is lost in cooking are the temperature, length of time if fried, and how often the oil is reused [3]. Duration and temperature of cooking affect vitamin A content, with lower temperatures and one or no reuse resulting in the highest retention.

# References

1. Dohlman E, Persaud S, Landes R (2003) India’s Edible Oil Sector: Imports Fill Rising Demand. USDA. OCS-0903-01 OCS-0903-01.

2. Shiva V (2001) The mustard oil conspiracy. The Ecologist 31: 27-29.

3. Nicholas Piramal India Limited (2006) "Human Nutrition & Health—Fortification." http://www.vfcdnicholas.com/hnh/fortification6.htm (accessed: November 15, 2006)
