# Supplementary material for: Cost-Effectiveness of “Golden Mustard” for Treating Vitamin A Deficiency in India
Source: PLoS One. 2010 Aug 10;5(8):e12046. doi: 10.1371/journal.pone.0012046 (PMC2919400; doi:10.1371/journal.pone.0012046)
Supplement: Notes S2 — Sensitivity analysis. (0.04 MB DOC) [file pone.0012046.s002.doc]

SUPPLEMENTARY NOTES

for

**Cost-Effectiveness of “Golden Mustard” for Treating**

**Vitamin A Deficiency in India**

Jeffrey Chow

Yale University

School of Forestry and Environmental Studies

New Haven, CT, USA

Eili Y. Klein

Princeton University

Department of Ecology and Evolutionary Biology

Princeton, NJ, USA

Ramanan Laxminarayan

Center for Disease Dynamics, Economics, and Policy

Washington, DC, USA

&

Princeton University

Princeton Environmental Institute

Princeton, NJ, USA

# Sensitivity Analysis

Because of the uncertainty surrounding our assumed parameters, we conducted a sensitivity analysis using Latin Hypercube Sampling (LHS) to evaluate the robustness of our results [1]. LHS, a type of stratified Monte Carlo sampling, efficiently analyzes large numbers of input parameters by treating each parameter as a separate random variable. A standard Monte Carlo simulation randomly selects each input parameter from within a probability distribution function. In LHS, each parameter distribution is stratified into equiprobable intervals and each interval is sampled exactly once, without replacement. An input vector is then generated, composed of the random samples of each of the input parameters for each simulation. The efficiency of LHS comes from each value of every parameter being used only once. The model may then be run *N* times to directly derive distribution functions for each of the outcome variables, and because of the probabilistic selection technique, the results can be interpreted within a statistical framework. Studies have shown that LHS is significantly more efficient than simple random and fractional stratified sampling designs [see 2], and is a commonly used method to evaluate models within the field of epidemiology [3-11].

Distributions of parameters were based on the number and range of observations describing the data. The effectiveness of an intervention in reducing the morbidity burden of VAD has numerous estimates across a range of values, so we assumed a uniform distribution across that range. Costs of fortification, both traditional and GM, were based on conservative and optimistic values, and so we again used a uniform distribution on the assumption that the costs were likely to be somewhere between the two values. The efficacy of VAD supplementation to reduce mortality has been contentious, as explained in the main text. As it had the most significant impact on the results no matter what distribution was assumed, we ran the sensitivity analysis for both the conservative estimate and the optimistic estimate and varied the other parameters at that efficacy level. Lastly, as we only had a single estimate for state specific prevalence of VAD morbidity, we assumed a triangular distribution which varied from 0 to twice the estimate to account for any sampling issues in the estimate.

# References

1. McKay MD, Beckman RJ, Conover WJ (1979) A comparison of three methods for selecting values of input variables in the analysis of output from a computer code. Technometrics 21: 239-245.

2. Blower SM, Dowlatabadi H (1994) Sensitivity and uncertainty analysis of complex models of disease transmission: an HIV model, as an example. International Statistical Review/Revue Internationale de Statistique 62: 229-243.

3. Sanchez MA, Blower SM (1997) Uncertainty and sensitivity analysis of the basic reproductive rate. Tuberculosis as an example. American Journal of Epidemiology 145: 1127-1137.

4. Blower SM, Porco TC, Darby G (1998) Predicting and preventing the emergence of antiviral drug resistance in HSV-2. Nature Medicine 4: 673-678.

5. Schuette MC, Hethcote HW (1999) Modeling the effects of varicella vaccination programs on the incidence of chickenpox and shingles. Bulletin of Mathematical Biology 61: 1031-1064.

6. Blower SM, Gershengorn HB, Grant RM (2000) A tale of two futures: HIV and antiretroviral therapy in San Francisco. Science 287: 650-654.

7. Blower SM, Koelle K, Kirschner DE, Mills J (2001) Live attenuated HIV vaccines: predicting the tradeoff between efficacy and safety. Proceedings of the National Academy of Sciences 98: 3618.

8. Blower S, Ma L (2004) Calculating the contribution of herpes simplex virus type 2 epidemics to increasing HIV incidence: treatment implications. Clinical Infectious Diseases 39: S240-S247.

9. Blower SM, Chou T (2004) Modeling the emergence of the ‘hot zones’: tuberculosis and the amplification dynamics of drug resistance. Nature Medicine 10: 1111-1116.

10. Schwartz EJ, Blower SM (2005) Predicting the potential individual level and population level impact of HSV-2 vaccines. Journal of Infectious Diseases 191: 1734-1746.

11. Currie CS, Williams BG, Cheng RC, Dye C (2003) Tuberculosis epidemics driven by HIV: is prevention better than cure. AIDS 17: 2501-2508.
