# Supplementary figures and images for: Cost-Effectiveness of “Golden Mustard” for Treating Vitamin A Deficiency in India
Source: PLoS One. 2010 Aug 10;5(8):e12046. doi: 10.1371/journal.pone.0012046 (PMC2919400; doi:10.1371/journal.pone.0012046)

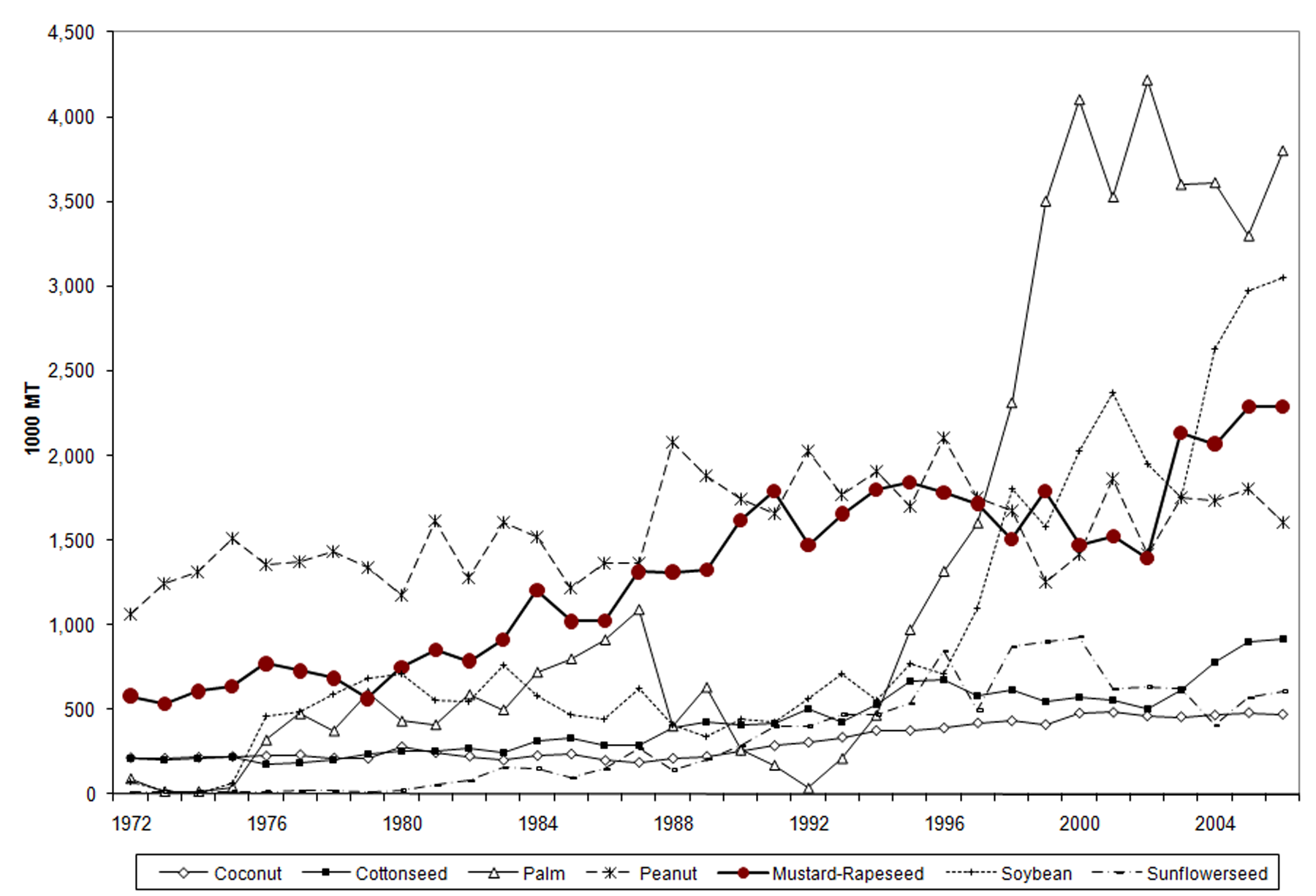

Supplement: Figure S1 — Total domestic consumption of edible oil, 1972–2006, by type. Note: Consumption for 2006 is an estimate. Source: United States Department of Agriculture 2006. (0.41 MB TIF) [file pone.0012046.s003.tif]

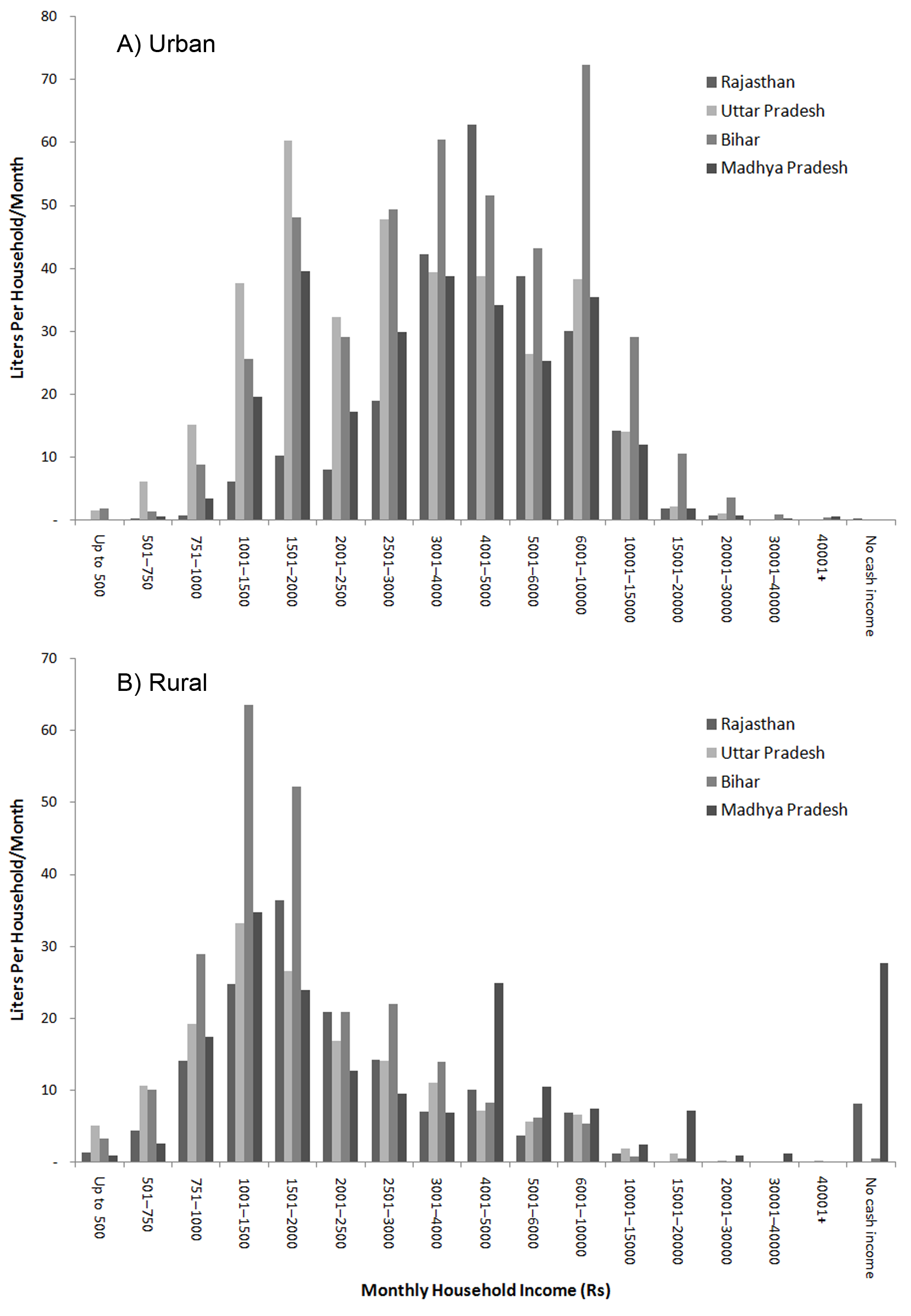

Supplement: Figure S2 — Monthly per capita purchases of mustard oil by household income in rural and urban households of Rajasthan, Uttar Pradesh, Bihar, and Madhya Pradesh, 2005. Source: IMRB International (2006). (2.84 MB TIF) [file pone.0012046.s004.tif]
