# Supplementary material for: Cost-Effectiveness of “Golden Mustard” for Treating Vitamin A Deficiency in India
Source: PLoS One. 2010 Aug 10;5(8):e12046. doi: 10.1371/journal.pone.0012046 (PMC2919400; doi:10.1371/journal.pone.0012046)
Supplement: Table S1 — Parameter values for sensitivity analysis. (0.11 MB DOC) [file pone.0012046.s005.doc]

Table S1: Parameter values for sensitivity analysis

| **Intervention Effectiveness in Reducing VAD burden^a^** | |
| --- | --- |
| Effectiveness against Bitot's Spot | 26%-75% |
| Effectiveness against Night blindness | 46%-100% |
| Effectiveness against Blindness | 43%-75% |
| **Fortification^a^** | |
| Cost of Fortification (Rs/kg) | $0.10-0.28 |
| Bottling Costs (Rs/kg) | $2.72-4.08 |
| **GM fortification** | |
| Bottling Costs (Rs/kg)^a^ | $0.28-4.08 |
| Fixed Costs (Rs/kg) ^b^ | $0.45 (0.36-0.57) |
| **Mortality^c^** | |
| Children (low) | 0.04 |
| Children (high) | 0.23 |
| Maternal (low) | 0.04 |
| Maternal (high) | 0.23 |
| **Bitot’s Spot Prevalence^b^** | |
| Uttar Pradesh | 5.8% (0-11.6) |
| Bihar | 1.8% (0-3.6) |
| Assam | 3.1% (0-6.2) |
| West Bengal | 0.6% (0-1.2) |
| Madhya Pradesh | 1.6% (0-3.2) |
| Andhra Pradesh | 1.2% (0-2.4) |
| Rajasthan | 1.1% (0-2.2) |
| Jharkhand | 4.4% (0-8.8) |
| Orissa | 0.3% (0-0.6) |
| Haryana | 0.3% (0-0.5) |
| Gujarat | 0.0% (0-0.0) |
| Punjab | 0.0% (0-0.1) |
| Jammu & Kashmir | 0.0% (0-0.1) |
| Himachal Pradesg | 0.1% (0-0.1) |
| Manipur | 0.1% (0-0.1) |
| Nagaland | 0.2% (0-0.5) |
| Tripura | 1.7% (0-3.4) |
| Delhi (urban) | 0.0% (0-0.0) |
| Chandigarh (urban) | 0.6% (0-1.2) |
| Uttar Pradesh (urban) | 2.4% (0-4.8) |
| Bihar (urban) | 0.0% (0-0.0) |
| West Bengal (urban) | 0.5% (0-1.0) |
| Madhya Pradesh (urban) | 3.8% (0-7.6) |
| Jharkhand (urban) | 10.7% (0-21.4) |
| Jammu & Kashmir (urban) | 0.0% (0-0.1) |
| **Night Blindness Prevalence (children)^b^** | |
| Uttar Pradesh | 0.8% (0-1.6) |
| Bihar | 1.5% (0-3.0) |
| Assam | 1.6% (0-3.2) |
| West Bengal | 0.7% (0-1.4) |
| Madhya Pradesh | 0.3% (0-0.6) |
| Andhra Pradesh | 0.4% (0-0.8) |
| Rajasthan | 0.1% (0-0.2) |
| Jharkhand | 0.8% (0-1.7) |
| Orissa | 1.1% (0-2.2) |
| Haryana | 0.3% (0-0.6) |
| Gujarat | 0.1% (0-0.2) |
| Punjab | 0.0% (0-0.0) |
| Jammu & Kashmir | 0.8% (0-1.7) |
| Himachal Pradesg | 0.0% (0-0.0) |
| Manipur | 0.1% (0-0.2) |
| Nagaland | 0.3% (0-0.6) |
| Tripura | 1.1% (0-2.2) |
| Delhi (urban) | 1.2% (0-2.4) |
| Chandigarh (urban) | 1.6% (0-3.2) |
| Uttar Pradesh (urban) | 3.6% (0-7.2) |
| Bihar (urban) | 1.5% (0-3.0) |
| West Bengal (urban) | 0.7% (0-1.4) |
| Madhya Pradesh (urban) | 0.3% (0-0.6) |
| Jharkhand (urban) | 0.8% (0-1.7) |
| Jammu & Kashmir (urban) | 0.8% (0-1.7) |
| **Night Blindness Prevalence (maternal)^b^** | |
| Uttar Pradesh | 15.2% (0-30.4) |
| Bihar | 20.2% (0-40.4) |
| Assam | 7.2% (0-14.4) |
| West Bengal | 13.1% (0-26.2) |
| Madhya Pradesh | 22.1% (0-44.2) |
| Andhra Pradesh | 6.3% (0-12.6) |
| Rajasthan | 15.5% (0-31.0) |
| Jharkhand | 11.4% (0-22.7) |
| Orissa | 19.3% (0-38.6) |
| Haryana | 0.9% (0-1.8) |
| Gujarat | 13.1% (0-26.2) |
| Punjab | 0.6% (0-1.2) |
| Jammu & Kashmir | 19.4% (0-38.8) |
| Himachal Pradesg | 3.9% (0-7.8) |
| Manipur | 0.0% (0-0.0) |
| Nagaland | 21.1% (0-42.2) |
| Tripura | 11.4% (0-22.7) |
| Delhi (urban) | 3.8% (0-7.6) |
| Chandigarh (urban) | 1.5% (0-2.9) |
| Uttar Pradesh (urban) | 7.5% (0-15.0) |
| Bihar (urban) | 11.7% (0-23.4) |
| West Bengal (urban) | 5.0% (0-10.0) |
| Madhya Pradesh (urban) | 10.7% (0-21.4) |
| Jharkhand (urban) | 11.4% (0-22.7) |
| Jammu & Kashmir (urban) | 13.9% (0-27.8) |
| **Mustard Consumption (Kg/Month/capita)^b^** | |
| Uttar Pradesh | 0.41 (0-0.82) |
| Bihar | 0.35 (0-0.70) |
| Assam | 0.34 (0-0.68) |
| West Bengal | 0.41 (0-0.82) |
| Madhya Pradesh | 0.12 (0-0.24) |
| Andhra Pradesh | 0.00 (0-0.00) |
| Rajasthan | 0.24 (0-0.48) |
| Jharkhand | 0.35 (0-0.70) |
| Orissa | 0.17 (0-0.34) |
| Haryana | 0.18 (0-0.36) |
| Gujarat | 0.03 (0-0.06) |
| Punjab | 0.09 (0-0.18) |
| Jammu & Kashmir | 0.63 (0-1.26) |
| Himachal Pradesg | 0.33 (0-0.66) |
| Manipur | 0.22 (0-0.44) |
| Nagaland | 0.20 (0-0.40) |
| Tripura | 0.58 (0-1.16) |
| Delhi (urban) | 0.36 (0-0.72) |
| Chandigarh (urban) | 0.20 (0-0.40) |
| Uttar Pradesh (urban) | 0.42 (0-0.84) |
| Bihar (urban) | 0.42 (0-0.84) |
| West Bengal (urban) | 0.58 (0-1.16) |
| Madhya Pradesh (urban) | 0.14 (0-0.28) |
| Jharkhand (urban) | 0.35 (0-0.71) |
| Jammu & Kashmir (urban) | 0.67 (0-1.34) |
| a: Uniform distribution used across range stated  b: Triangular distribution used with mode value stated and min and max values listed in parentheses  c: Fixed values | |

### 
