# Supplementary material for: Cost-Effectiveness of “Golden Mustard” for Treating Vitamin A Deficiency in India
Source: PLoS One. 2010 Aug 10;5(8):e12046. doi: 10.1371/journal.pone.0012046 (PMC2919400; doi:10.1371/journal.pone.0012046)
Supplement: Table S2 — State-specific cost-effectiveness calculations ($/DALY averted). (0.05 MB DOC) [file pone.0012046.s006.doc]

### Table S2: State-specific cost-effectiveness calculations ($/DALY averted)

| **Rural Areas** | **Supplementation** | **Fortification** | **GM fortification** |
| --- | --- | --- | --- |
| Uttar Pradesh | 12–9 | 230-410 | 130-230 |
| Bihar | 24–15 | 210-350 | 110-180 |
| Assam | 15–10 | 230-450 | 110-220 |
| West Bengal | 54–31 | 510-850 | 280-480 |
| Madhya Pradesh | 20–11 | 140-260 | 50-90 |
| Rajasthan | 33–19 | 230-410 | 100-180 |
| Jharkhand | 14–10 | 300-690 | 140-330 |
| Orissa | 29–14 | 140-270 | 60-120 |
| Haryana | 56–27 | 430-940 | 170-360 |
| Gujarat | 59–26 | 320-540 | 100-160 |
| Punjab | 72–32 | 480-1,090 | 160-360 |
| Jammu & Kashmir | 46–20 | 350-600 | 260-440 |
| Himachal Pradesh | 96–44 | 750-1,520 | 410-840 |
| Manipur | 34–15 | 290-660 | 120-270 |
| Nagaland | 31–14 | 220-380 | 90-150 |
| Tripura | 21–12 | 340-740 | 250-550 |
| **Pooled** | 20–13 | 180-260 | 90-210 |
|  |  |  |  |
| **Urban Areas** |  |  |  |
| Delhi | 80–40 | 630-1,170 | 310-590 |
| Chandigarh | 59–37 | 780-1,450 | 300-570 |
| Uttar Pradesh | 22–18 | 420-690 | 240-390 |
| Bihar | 20–15 | 400-700 | 210-380 |
| West Bengal | 84–56 | 1,680-2,900 | 1,140-1,960 |
| Madhya Pradesh | 17–14 | 450-800 | 170-300 |
| Jharkhand | 7–7 | 720-1,670 | 360-830 |
| Jammu & Kashmir | 90–40 | 820-1,350 | 600-990 |
| **Pooled** | 25–19 | 410-580 | 220-420 |
| Range provided is for low mortality (conservative) and high mortality (optimistic) reduction scenarios. | | | |
